# Methods for detecting gene × gene interaction in multiplex extended pedigrees

**DOI:** 10.1186/1471-2156-6-S1-S144

**Published:** 2005-12-30

**Authors:** Guy N Brock, Brion S Maher, Toby H Goldstein, Margaret E Cooper, Mary L Marazita

**Affiliations:** 1 Department of Human Genetics, University of Pittsburgh, 130 Desoto St., Pittsburgh, PA 15261 USA; 2 School of Dental Medicine, University of Pittsburgh, 130 Desoto St., Pittsburgh, PA 15261 USA

## Abstract

Complex diseases are multifactorial in nature and can involve multiple loci with gene × gene and gene × environment interactions. Research on methods to uncover the interactions between those genes that confer susceptibility to disease has been extensive, but many of these methods have only been developed for sibling pairs or sibships. In this report, we assess the performance of two methods for finding gene × gene interactions that are applicable to arbitrarily sized pedigrees, one based on correlation in per-family nonparametric linkage scores and another that incorporates candidate loci genotypes as covariates into an affected relative pair linkage analysis. The power and type I error rate of both of these methods was addressed using the simulated Genetic Analysis Workshop 14 data. In general, we found detection of the interacting loci to be a difficult problem, and though we experienced some modest success there is a clear need to continue developing new methods and approaches to the problem.

## Background

The topic of gene × gene interaction (epistasis) has recently elicited great interest [1,2] and even controversy [3,4] in the literature. The reason for the increasing attention to methods to detect epistasis is primarily the general lack of success in finding single gene contributions to complex disease risk. Consequently, approaches to detecting interaction have been developed within the framework of both linkage and association analysis. Some methods focus on detecting interaction with a particular candidate or target locus [5], while others are more exploratory in nature (i.e., not motivated by a marginal significance) [6]. In this report, we investigate methods for detecting interacting loci within a linkage analysis using the simulated Genetic Analysis Workshop 14 (GAW14) data, particularly focusing on procedures that are applicable to large, extended pedigrees.

Many approaches to detecting genetic interactions using linkage analysis, both parametric and nonparametric, have been proposed. For reasons discussed by Cox et al. [5], we will focus our discussion on nonparametric methods, particularly those that are applicable to extended pedigrees. The approach of Cox et al. [5] focuses on examining the correlation between per-family linkage statistics. First suggested by MacLean et al. [7], positive correlation between linkage statistics at unlinked regions of the genome is indicative of statistical interaction between those regions. This approach was later used to detect interaction between multiple loci in non-insulin dependent diabetes mellitus [8] under a parametric linkage model. The nonparametric linkage (NPL) correlation approach is especially useful when a locus of interest due to a linkage signal has been identified but a candidate gene (marker) has not yet been characterized.

A slightly different scenario is that in which a candidate marker has been identified and one wishes to incorporate that marker into the linkage analysis. One approach that can be used for such an analysis in large multiplex pedigrees is a re-parameterized version of Risch's [9] LOD score model for affected relative pair linkage as implemented in the LODPAL program of the Statistical Analysis for Genetic Epidemiology (S.A.G.E.) package [6,10]. LODPAL models the relative risk for arbitrary sets of affected relative pairs, and is therefore applicable to extended pedigrees. To test for genetic interactions, models can be tested with candidate loci (e.g., single-nucleotide polymorphism (SNP) genotypes) included as covariates. Significant interactions are evidenced by increases in the linkage signal due to the inclusion of the covariate.

## Methods

We investigated the correlation-based approach suggested by Cox et al. [5] and the covariate-based approach implemented in LODPAL [6] for detecting genetic interactions in a linkage framework. For the correlation-based approach we investigated potential interactions, indexed as positive correlations in linkage statistics, with selected markers in the vicinity of the disease loci. For the covariate-based approach using LODPAL we included candidate marker genotypes from the original SNP panel as covariates in a nonparametric linkage analysis.

In order to investigate the power and type I error of both approaches, we used the simulated GAW14 data. For a complete description of the simulated datasets, see [11]. In brief, family data were collected from four populations (Aipotu (AI), Karangar (KA), Danacaa (DA), and NYC (NY)). The AI, KA, and DA studies included only nuclear families, while the NY study ascertained mostly three-generation pedigrees. For our purposes, the NY pedigrees were particularly relevant because they were multigenerational, although for the correlation-based method the AI, KA, and DA families were also analyzed. The families from all four studies were ascertained on the basis of three underlying latent traits, and the disease phenotype (Kofendrerd Personality Disorder, or KPD) was diagnosed based on the presence of at least one of the underlying latent traits. The genetic etiology of each latent trait was distinct and involved interactions between several disease loci. We consulted the answers prior to our study to identify these existing interactions, and selected markers to use as candidate loci based on the locations of each of the disease loci D1–D4. Because the underlying latent traits P1–P3 contained clearly defined genetic interactions, we included these traits in our analysis in addition to the KPD phenotype.

Type I error for each method was indicated by the proportion of times a significant interaction was found with marker loci on the null chromosomes (chromosomes that lacked any disease loci), while the power for each method was assessed by the proportion of times a significant interaction was found with the marker locus closest to the interacting disease locus.

### Detection of epistasis in linkage analysis – correlation approach

Multipoint NPL scores were calculated using MERLIN for the KPD disease phenotype and the three latent traits in the KA, DA, and AI populations. Single-point calculations were performed for the NY population. In all cases the microsatellite data were used. For candidate loci, the four microsatellite markers nearest to the disease loci D1, D2, D3, and D4 were selected. The correlations in NPL scores between these candidate loci and the remaining microsatellite markers were then calculated. A significant positive correlation (α = 0.05) indicated a potential epistatic interaction between the two loci, while a significant negative correlation indicated potential heterogeneity. When a significant correlation was detected, NPL scores were then re-calculated using the per-family NPL scores of the candidate locus.

All of the markers on chromosomes 4, 6, 7, and 8 were used to estimate the empirical type I error rate. These chromosomes (the null chromosomes) were selected because they lacked any disease loci. For each combination of phenotype and population, the proportion of markers on the null chromosomes having significant positive correlations (one-sided *p*-value < 0.05) with the four candidate loci was estimated using all 100 simulations. The power of the method was assessed using the proportion of times a significant positive correlation was detected between two interacting candidate loci (again using a 0.05 significance level).

### Weighted NPL analysis

For prominent interactions, we tested whether conditioning on the family-wise NPL scores of one disease locus would improve the overall NPL score of the interacting locus. Families were given a 0–1 weighting depending on whether the NPL score of the conditioning locus was negative or positive, respectively, and the NPL scores were re-calculated with these weights using ALLEGRO 1.2 [12].

### Detection of epistasis in linkage analysis – covariate approach

In this method, the marker data were used to estimate the proportion of alleles identical by descent (IBD) at each polymorphic marker locus for pairs of affected relatives. Multipoint IBD-sharing estimates for the NY pedigrees were obtained using the GENIBD program from the S.A.G.E. package, release 4.3 [10]. Exact estimates were calculated for pedigrees where 2*n*-*f *< 18, where *n *and *f *are the number of nonfounders and founders in the pedigree, respectively. For pedigrees where 2*n*-*f *> 18, a modified Markov chain Monte Carlo (MCMC) simulation algorithm [13] was used to generate IBD estimates. For direct comparison with the correlation-based approach, single-point IBD estimates were also calculated.

For these analyses we used a modified one-parameter model of the conditional logistic model [14], with candidate loci (the SNP closest to each disease locus) included as covariates. Both dominant and recessive phenotypes were modelled for the genotypes of each SNP, and the covariate was an indicator of agreement between the SNP phenotypes for each affected relative pair.

Linkage parameters were first estimated under the base model without any covariates and then recalculated with each candidate locus included as a covariate, i.e., the genome scan was repeated for each covariate. Regions in which the LOD score with a covariate was significantly increased versus the base model were presumably regions with genes that interacted with the candidate locus (covariate). To address concerns that the behavior of the test statistic, when applied to large multiplex families, was influenced by both family and marker characteristics, we performed the analysis for each model (each SNP covariate and phenotype) on the null chromosomes in the simulated dataset to determine the null distribution of the test statistic when no disease locus was present. Empirically, the mean (SD) inflation of the LOD difference statistic under the null ranged from 0.28 (0.39) to 0.42 (0.59) across all models tested. The power of the method was determined by the proportion of times the change in LOD score was above the 95^th ^percentile of the empirical null distribution.

## Results

### Correlation approach

The results for the empirical type I error rates of the correlation method are given in Table 1. The empirical error rates were consistently above the nominal rates, with a high of 0.0848 for the DA population and the KPD and P1 phenotypes. This indicates that significance levels based on the normal distribution will give liberal results, and suggests using a more stringent cut-off or empirical estimates of significance.

Table 2 gives the power for detecting significant correlations between interacting loci using the correlation approach. The best results were for the D1–D4 interaction in the P3 latent trait for the AI, KA, and NY populations and the D1–D2 interaction in the P1 and KPD phenotype for the DA population. The power to detect the other interactions between loci was considerably less. The lower power could be due to one of several reasons, including a lower penetrance for the multilocus genotype, reduced sample sizes when analyzing the latent traits, and conflicting relations between the loci (i.e., heterogeneity vs. epistasis) when analyzing the KPD phenotype.

### Weighted NPL analysis

To illustrate, we show results from the D1–D4 interaction in the P3 phenotype. When restricting attention to replicates with a significant positive correlation between D1 and D4 (otherwise the conditioning is pointless), the average increase in NPL scores was 0.549 and 0.638 for the AI and KA populations, respectively, when conditioning on the D4 locus and 0.354, and 0.444 when conditioning on the D1 locus. This increase in the NPL score is highly correlated (*r = *0.703) with the degree of correlation between the two loci. Also, the effectiveness of re-calculating the NPL score is highly dependent upon which locus is conditioned upon. In this case, conditioning upon the D4 locus is more effective than conditioning on the D1 locus due to the stronger LD in the D4 region.

### Covariate approach

Results for the power of the conditional logistic model, which includes the closest SNP to the disease locus as a covariate, are given for the NY population for the KPD phenotype and each of the three latent traits. Power for the approach, using a significance level of 0.05, is presented in Table 3. In general, the conditioning approach using LODPAL outperformed the correlation-based method. Overall, the results are modest but the best results occurred when the interactions were the simplest (just epistasis; not epistasis and heterogeneity). Also, the model appeared fairly robust towards misspecification of the SNP parameterization (dominant vs. recessive). As in the weighted NPL analysis, conditioning on the SNP in the strongest linkage disequilibrium with the disease locus proved to be the most effective. For example, in the P1 trait the power is much greater when conditioning on the D2 locus, and in the P3 trait the power is much greater when conditioning on the D4 locus.

## Discussion

Overall, detecting genetic interactions using correlations in NPL scores or by conditioning on candidate genotypes proved to be difficult. The conditioning approach was more successful, but in both cases the best results occurred when analyzing the latent traits separately or when the sample was relatively homogeneous, as in the KA and DA studies. When the disease phenotype is a heterogenous mixture of latent traits, both heterogeneity and epistasis can exist between the disease loci among the different traits, making detection of the interaction extremely difficult. One possible solution is to use principal components or factor analysis to potentially resolve these underlying latent variables. These latent traits can be analyzed directly using model-free methods that incorporate interaction such as variance components [15], Haseman-Elston regression (and its extensions) [16], and score statistics [17].

Because LODPAL treats all affected relative pairs in a pedigree as independent, the linkage signals obtained from LODPAL will tend to be over-inflated. In our experience, we also found that the difference in linkage scores when a covariate was included was over-inflated. Therefore, it is necessary to determine the significance level of this change in linkage score empirically. Our approach was to use the null chromosomes in the simulated data to generate an empirical distribution. However, this method does not condition on the observed sharing at the marker being tested, and an alternative approach is to permute the covariate values. Interestingly, the number of positive correlations between the candidate loci and unrelated loci was also in excess of what was expected, revealing a high type I error rate. An empirical distribution could be generated by randomly selecting a subset of markers and then permuting their per-family NPL scores multiple times. Approximate significance levels could then be obtained with this empirical distribution.

Testing multiple models with higher order interaction terms poses a serious scaling problem that needs to be addressed. One way to alleviate this problem is to incorporate existing information concerning biological pathways and interactions to restrict the number of models being tested. However, because linkage studies are generally exploratory in nature, there may be little information for many genomic regions of interest. Most likely, there are no easy answers for unravelling the genetic interactions that exist in complex diseases. Given that information regarding biological interaction will not generally be available, new approaches to detecting epistasis in extended pedigrees must be examined.

## Abbreviations

GAW14: Genetic Analysis Workshop 14

IBD: Identical by descent (IBD)

KPD: Kofendrerd Personality Disorder

MCMC: Markov chain Monte Carlo

NPL: Nonparametric linkage

SNP: Single-nucleotide polymorphism

## Authors' contributions

All authors participated in the design of the study. GNB, BSM, THG, MEC, performed the statistical analyses. MLM acted as a supervisor for the design and statistical analyses. GNB and BSM drafted the manuscript. All authors read and approved the final manuscript.

## Figures and Tables

**Table 1:** Type I error rates for significant positive correlations

	Population			
	
Phenotype	AI^a^	KA^a^	DA^a^	NY^b^
KPD	0.0793	0.0792	0.0848	0.071
P1	0.0703	0.0686	0.0848	0.0696
P2	0.0711	0.0793	0.0531	0.0714
P3	0.0635	0.0686	0.0531	0.0595

^a ^multipoint estimates

^b ^single-point estimates

Error rates are based on the proportion of times a disease locus had a significant positive correlation (one-sided *p*-value < 0.05) with the loci on chromosomes 4, 6, 7, and 8.

**Table 2:** Power for detecting significant correlations between interacting loci

		Population			
		
Phenotype	Disease loci	AI^a^	KA^a^	DA^a^	NY^b^
KPD	D1 and D2	0.11	0.05	0.28	0.09
	D1 and D4	0.06	0.2	0.04	0.13
	D2 and D3	0.1	0.14	0.1	0.07
	D3 and D4	0.07	0.02	0.07	0.07
P1	D1 and D2	0.12	-	0.28	0.07
P2	D2 and D3	0.17	0.14	-	0.1
	D3 and D4	0.01	0.02	-	0.01
P3	D1 and D4	0.26	0.24	-	0.16
	D2 and D3	0.12	0.11	-	0.06

^a ^multipoint estimates

^b ^single-point estimates

Power is based on the proportion of times a positive correlation between two loci had a one-sided *p*-value < 0.05. Based on 100 replicates.

**Table 3:** Power for detecting interactions between loci using LODPAL (NY population)

Phenotype	SNP covariate	Model	Marker-region	Power (single point)	Power (multipoint)
KPD	D1	dom	D2	0.23	0.20
		rec	D2	0.18	0.22
		dom	D4	0.19	0.17
		rec	D4	0.14	0.11
	D2	dom	D1	0.29	0.22
		rec	D1	0.25	0.21
		dom	D3	0.22	0.19
		rec	D3	0.15	0.10
	D3	dom	D2	0.26	0.23
		rec	D2	0.17	0.15
		dom	D4	0.20	0.20
		rec	D4	0.12	0.14
	D4	dom	D1	0.32	0.26
		rec	D1	0.25	0.20
		dom	D3	0.23	0.23
		rec	D3	0.15	0.13

P1	D1	dom	D2	0.14	0.10
		rec	D2	0.18	0.14
	D2	dom	D1	0.42	0.29
		rec	D1	0.32	0.34

P2	D2	dom	D3	0.21	0.18
		rec	D3	0.13	0.09
	D3	dom	D2	0.22	0.14
		rec	D2	0.11	0.10
		dom	D4	0.16	0.17
		rec	D4	0.18	0.13
	D4	dom	D3	0.18	0.21
		rec	D3	0.16	0.12

P3	D1	dom	D4	0.14	0.09
		rec	D4	0.08	0.07
	D2	dom	D3	0.14	0.08
		rec	D3	0.11	0.08
	D3	dom	D2	0.09	0.13
		rec	D2	0.12	0.09
	D4	dom	D1	0.25	0.19
		rec	D1	0.17	0.18
